# Grape exosome-like nanoparticles: A potential therapeutic strategy for vascular calcification

**DOI:** 10.3389/fphar.2022.1025768

**Published:** 2022-10-21

**Authors:** Yintong Teng, Jiaqi He, Qingping Zhong, Yangmei Zhang, Zhenxing Lu, Tianwang Guan, Yuxuan Pan, Xiaodi Luo, Weijing Feng, Caiwen Ou

**Affiliations:** ^1^ Department of Cardiology, Guangdong Provincial Key Laboratory of Shock and Microcirculation, Dongguan Hospital of Southern Medical University, Southern Medical University, Guangzhou, China; ^2^ Department of Cardiology, Laboratory of Heart Center, Zhujiang Hospital, Southern Medical University, Guangzhou, China; ^3^ Department of Cardiothoracic Surgery, 920th Hospital of Joint Logistics Support Force of People’s Liberation Army of China, Kunming, China; ^4^ Department of Cardiology, State Key Laboratory of Organ Failure Research, Guangdong Provincial Key Lab of Shock and Microcirculation, Nanfang Hospital, Southern Medical University, Guangzhou, China

**Keywords:** grape exosome-like nanoparticles, vascular calcification, vascular smooth muscle cells, exosomes, osteogenic phenotype differentiation

## Abstract

Vascular calcification (VC) is prevalent in hypertension, diabetes mellitus, chronic kidney disease, and aging and has been identified as an important predictor of adverse cardiovascular events. With the complicated mechanisms involved in VC, there is no effective therapy. Thus, a strategy for attenuating the development of VC is of clinical importance. Recent studies suggest that grape exosome-like nanoparticles (GENs) are involved in cell–cell communication as a means of regulating oxidative stress, inflammation, and apoptosis, which are known to modulate VC development. In this review, we discuss the roles of GENs and their potential mechanisms in the development of VC.

## Introduction

Vascular calcification (VC) is a pathological process characterized by abnormal deposition of hydroxyapatite (HA) crystals in the arterial intima or media of vascular walls, which can result in increased vascular stiffness and decreased vascular compliance (Nicoll and Henein, 2014; Bryan and Simon, 2015; Lanzer et al., 2021). VC commonly occurs in patients with chronic kidney disease (CKD), diabetes, and aging, and it is significantly associated with an increased risk of cardiovascular morbidity and mortality in these populations. The formation of VC is a complex and highly regulated pathological process, similar to bone development and chondrogenesis (Nakahara et al., 2017). Previous research has reported that VC is caused by hyperphosphatemia (Lee et al., 2020) and other risk factors including inflammation, oxidative stress, lipid deposition, and apoptosis. Despite its global clinical burden, no effective therapies are available to deal with VC due to its complicated underlying mechanisms.

Compelling epidemiological evidence suggests that fruit and vegetable consumption can improve lipid metabolism and endothelial function (Luc et al., 2004), lower blood pressure (John et al., 2002), and reduce oxidative stress (John et al., 2002; Zino et al., 1997). Plant-derived exosome-like nanoparticles (PDENs) were isolated and purified from plants that have lipid bilayers and functional cytosolic components such as mRNA, miRNA, proteins, and plant-homologous bioactive small molecules that can protect against vascular disease and cardiovascular-related mortality (Lichtenstein and Russell, 2005; Zhang et al., 2016a). Therefore, PDENs have been widely used to treat a variety of conditions (Mohadeseh et al., 2022) including pneumonia (Yun et al., 2021), intestinal inflammatory disease (Mu et al., 2016), cutaneous wounds (Yağız et al., 2021), and tumors (Meng et al., 2019). The therapeutic utility of PDENs is based on their anti-inflammatory, anti-oxidative, and anti-apoptotic properties. Grape exosome-like nanoparticle (GEN) is one of the most important PDENs. In this context, we consider GENs and discuss their potential role in VC prevention and treatment.

## GENs

### Molecular composition of GENs

**FIGURE 1:**
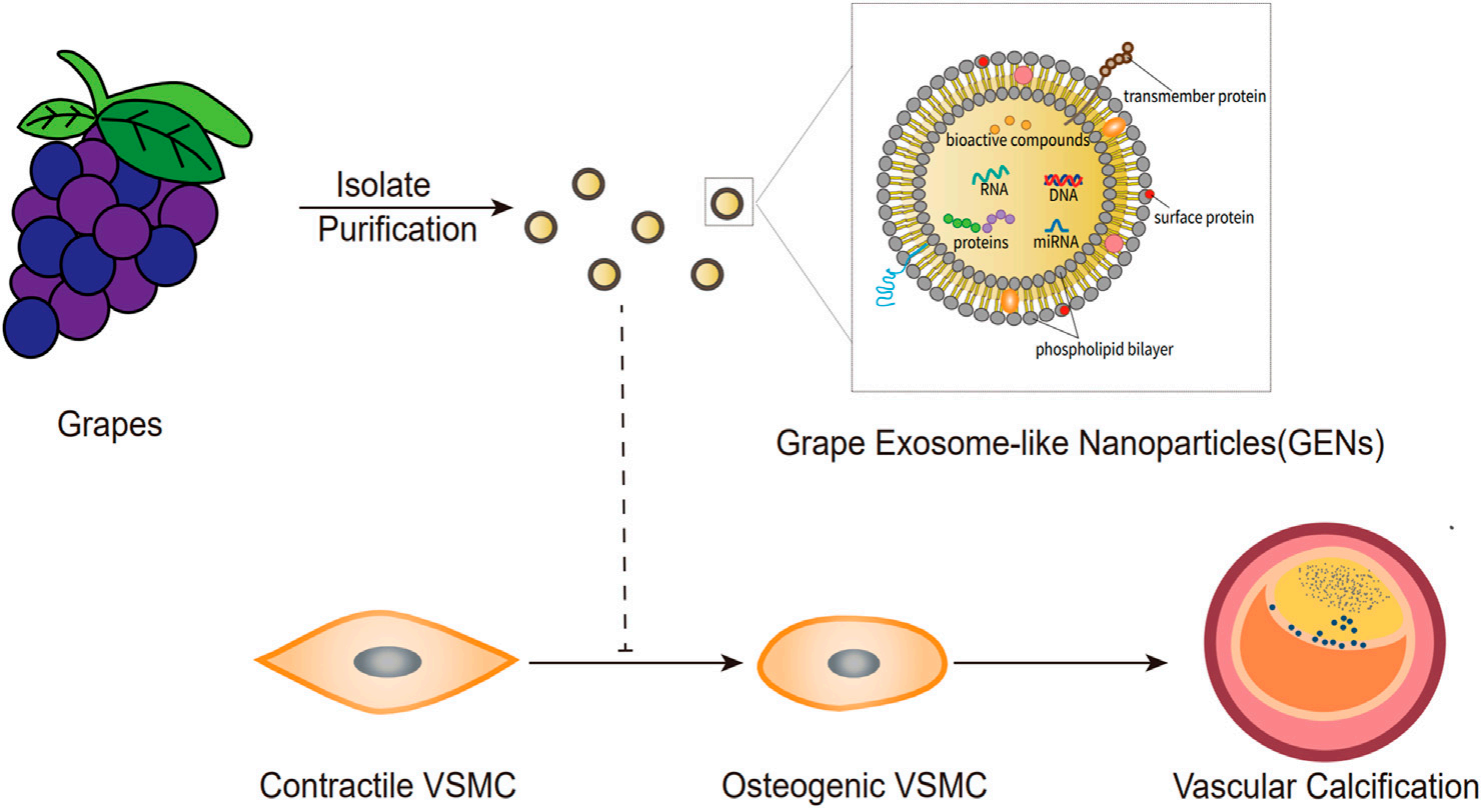
Structure of grape exosome-like nanoparticles (GENs) served as a bioactive vesicle for inhibiting the osteogenic differentiation of vascular smooth muscle cells.

GENs derived from grapes, including proteins, lipids, RNA (mRNAs, miRNAs, and lncRNAs), and natural small molecular compounds, can be transferred to recipient cells and exert biological effects, acting as messengers in intercellular or cross-species communication to treat disease by regulating biological functions (Hadi et al., 2007; Baomei et al., 2014; Doyle and Wang, 2019). Few studies have clarified the compositions and mechanisms of proteins in GENs. A previous study indicated that the proteins in GENs are similar to those found in animal-derived exosomes, with a lower protein content (Songwen et al., 2013). GENs were found to contain proteins that regulate glucose and lipid metabolism (Songwen et al., 2013). Many studies have indicated the presence of phosphatidylethanolamine, phosphatidylcholine, phosphatidic acid, digalactosyl diacylglycerol, monogalactosyl diacylglycerol, phosphatidylinositol, and phosphatidylserine in GENs. However, compared with exosomes in animal cells, GENs contain almost no cholesterol (Songwen et al., 2013; Qilong et al., 2016). MiRNAs in GENs are mostly from the miR169 family, which shares two sequences with human miRNAs (hsa-miR-4480 and hsa-miR-4662a-5p). Further research has revealed that some miRNAs can directly target the expression of inflammatory factor genes such as IL-6, IL-2, IL-5, and IL-1 (Songwen et al., 2013). *In vitro* data have indicated that miRNAs from GENs can specifically bind to mammalian miRNAs, affecting many important biological processes (Juan et al., 2018). In addition, natural small molecular compounds from grapes were found in GENs, such as procyanidin, polyphenol, and ACH09 **(** Figure 1; Table 1 **)**.

**TABLE 1:** Bioactive small molecules of GENs and their effects on the vascular system.

Small molecules of GENs	Functions	Reference
Procyanidin	Attenuation of oxidative damage and apoptosis	El-Andaloussi et al. (2012)
	Regulation of glucose metabolism: prevention of pancreatic dysfunction and preservation of a higher glucagon/insulin ratio	Downing et al. (2017), Rodríguez et al. (2022), and Grau-Bové et al. (2020)
	Regulation of lipid metabolism: inhibition of adipogenesis and stimulation of lipolysis	Wei S et al. (2018)
	Retardation of glycated low-density lipoprotein induced-cardiomyocyte apoptosis	Li et al. (2019)
	Inhibition of AGE-induced proliferation and migration of VSMCs	Cai et al. (2011)
	Anti-inflammatory effects	Yin et al. (2015) and Liu et al. (2020)
Flavonoids based on the C_6_-C_3_-C_6_ skeleton and the non-flavonoids	Modulation of the intestinal microbiota	Lu et al. (2021) and Rasines-Perea and Teissedre (2017)
	Regulation of cholesterol and lipoprotein metabolism	Myers et al. (2009)
	Inhibition of LDL oxidation and attenuation of the development of atherosclerosis	Magyar et al. (2012) and Miyagi et al. (1997)
	Anti-inflammatory effects	Albers et al. (2004)
	Inhibition of platelet activation and aggregation	Pace-Asciak et al. (1996) and Keevil et al. (2000)
	Inhibition of digestion enzymes, improvement of insulin resistance, and inhibition of advanced glycation end product (AGE) formation	Rasines-Perea and Teissedre (2017), Xiao and Högger (2014), Chen and Jiang (2016), and Cao et al. (2015)
ACH09	Prevention of oxidative stress and enhanced activity of the superoxide dismutase	da Costa et al. (2020)
	Improvement of insulin resistance and increase of GLUT-4	Santos et al. (2017)
	Amelioration of hypertension	da Costa et al. (2020)
	Reversion of increases in adiposity, plasma triglyceride levels, and glucose levels	Resende et al. (2013)
Polygalloyl polyflavan-3-ols	Inhibition of human platelet aggregation	Shanmuganayagam et al. (2012) and Nassiri-Asl and Hosseinzadeh (2016)
	Amelioration of low-density lipoprotein oxidation	Shanmuganayagam et al. (2012)
Myricetin	Lesser degree of cellular infiltration	Nassiri-Asl and Hosseinzadeh (2016) and Tiwari et al. (2009)
	Slowing the development of high blood pressure and reversion of metabolic alterations	Godse et al. (2010)
	Increase the levels of antioxidant agents	Borde et al. (2011)
Resveratrol	Amelioration of low-density lipoprotein oxidation and reduction of lipid peroxidation	Breuss and Atanasov (2019)
	Inhibition of cyclooxygenase-1 (COX-1), COX-2, and nuclear factor-κB(NF-κB)	Kundu et al. (2006), Yeung et al. (2004), and Meng et al. (2021)
	Increase in the formation of vasculoprotective nitric oxide (NO)	Leikert et al. (2002) and Wallerath et al. (2002)
	Suppression of advanced glycation end product (AGE)-induced proliferation of VSMCs	Mizutani et al. (2000)
	Inhibition of platelet aggregation	Wang et al. (2002) and Pace-Asciak et al. (1995)

### Biological characteristics of GENs

Small vesicles derived from plants were first observed with a bilayer membrane structure by electron microscopy in 1968 (Marchant and Robards, 1968). PDENs have been identified and isolated in numerous plants over decades of research and purified and confirmed using characterization techniques such as dynamic light scattering, zeta potential, and Western blotting (Nader et al., 2021). The average diameter of the GEN population was 37.47–380.5 nm. Zeta potential measurements indicated that GENs have a negative zeta potential ranging from -69.6 mV to +2.52 mV, with an average potential of -26.3–8.14 mV (Wenbo et al., 2021). GENs can maintain particle size and surface charge stability at physiological temperatures (37°C). In addition, GENs can exhibit significant stability after being stored in deep-freeze conditions (−80°C) for half a year. Biological molecules’ integrity of GENs can withstand repeated freeze–thaw cycles in the laboratory. GENs are smaller and more homogeneous in simulated gastric fluid and simulated intestinal fluid, with a lower zeta potential (Wenbo et al., 2021). GENs can be taken up by multiple cells including endotheliaocytes, lymphocytes, macrophagocytes, and others *via* the endocytic pathway, and the findings indicated that GEN uptake by recipient cells is energy-dependent. F4/80 macrophages and stem cells in the intestine consume GENs (Songwen et al., 2013). The cytochalasin D and the macrolide antibiotics, bafilomycin A1 and concanamycin A, which are highly specific V-ATPase inhibitors, significantly inhibit GEN uptake (Songwen et al., 2013). PDEN distribution *in vivo* varies depending on the route of administration. For example, GENs were found primarily in the distal small intestine, cecum, and colon after passing through the stomach and proximal small intestine after oral administration (Songwen et al., 2013). 1,1-Dioctadecyl-3,3,3,3-tetramethylindotricarbocyaine iodide (DiR) fluorescent signals are predominantly detected in the liver, lungs, kidneys, and splenic tissues 72 h after a tail vein or intraperitoneal injection, whereas intramuscular injections of DiR-labeled grapefruit-derived nanovectors (GNVs) are predominantly localized in the muscle. The majority of DiR-labeled GNVs were found in the lungs and brain after intranasal administration. No signal was found in lung tissue 72 h after intranasal administration (Qilong et al., 2016) (Figure 1).

### Applications of GENs as natural carriers

Similar to artificial liposomes, GENs are capable of delivering drugs, siRNA, DNA, and proteins to various types of cells, and the specificity of GEN is able to be increased by introducing targeting ligands for increasing curative effect (Zhang et al., 2016). Compared with artificial carriers, GENs possess high biocompatibility, low immunogenicity, and easy modifiability, which could cross the blood–brain barrier but could not pass through the placental barrier. Thus, GENs have better security and bright clinical applied prospect as drug carriers.

At present, the common methods of loading nucleic acid therapeutic agents in animal-derived exosomes adopt cell transfection and then collect agent-loaded exosomes (El-Andaloussi et al., 2012). By contrast, there are many methods of loading drugs in PDENs including co-incubation, sonication, repetitive freeze–thawing, and electroporation that provide convenience for drug loading (Cong et al., 2022).

### GEN isolation and purification

Isolation and purification of GENs are similar to those of exosomes in animal cells or body fluids. A combination of differential centrifugation and sucrose density-gradient ultracentrifugation is the “gold standard” to extract abundant GENs in a short time (Zhang et al., 2016). First, grapes are ground in a blender to extract juice and the juice is strained through a sieve or a piece of gauze. Next, differentially centrifugation (500 g for 10 min, 2,000 g for 20 min, 5,000 g for 30 min, and 10,000 g for 1 h) is used to remove large grape fibers, and then supernatant ultracentrifugation is used to concentrate GENs at 100,000 g for 2 h. Finally, for purification of the GENs, the concentrated solutions of GEN are transferred to a discontinuous sucrose gradient [8%, 30%, 45%, and 60% (g/v)] and ultracentrifuged at 150,000 g for 2 h to remove other vesicles and aggregates of proteins or RNA. The bands in the 30% and 45% layers are purified GENs. In addition, GEN concentration is determined by measuring the protein concentration using a BCA protein quantification assay kit (Songwen et al., 2013; Wenbo et al., 2021). GENs allow for large-scale production as they are plentiful and inexpensive.

## GENs: potential mechanisms for VC therapy

### Oxidative stress

The occurrence and development of VC are influenced by oxidative stress for two primary reasons: 1. increased cellular endogenous reactive oxygen species (ROS) levels; 2. an imbalance in ROS generation and ROS scavenging. Excessive ROS causes oxidative damage to DNA (Hongmei et al., 2010; Roberto et al., 2012), lipids, and proteins, inducing phenotypic transformation of vascular smooth muscle cells (VSMCs) by activating the *Akt* signaling pathways and upregulating *Runx2*, an essential osteogenic transcription factor (Ting et al., 2021). Excess oxidized low-density lipoprotein (OxLDL) hastens VSMC transdifferentiation into osteoblast-like cells (Zhang et al., 2016b). Diabetes, in particular, has been shown to play a synergistic role in VC *via* the advanced glycation end product and advanced glycation end product receptor, further increasing the correlation between hyperglycemia and oxidase stress from NADPH oxidase (Hongmei et al., 2010; Mody et al., 2001).

GENs were reported to be functional nanoparticles with the ability to scavenge ROS and protect against non-specific cell damage caused by ROS (Songwen et al., 2013). Zhang et al. synthesized polyphenol nanoparticles from grape seeds with adjustable size, excellent biocompatibility, and ROS scavenging capacity. The role of nanoparticles in preventing cell damage caused by ROS, accelerating wound healing, inhibiting ulcerative colitis, and regulating oxidative stress in dry eye syndrome has been validated (Songwen et al., 2013). Wang et al. created a drug delivery system based on grapeseed extract loaded with solid lipid nanoparticles to improve bioavailability and aqueous solubility compared with the parent compound. They discovered that nanoparticles reduce oxidative stress in respiratory epithelia and are anti-inflammatory and anti-apoptotic (Tianyou et al., 2021). Polygalloyl polyflavan-3-ols were reported to be associated with the cardiovascular disease-protective effects of grapes as they inhibit platelet aggregation and oxidation of OxLDL (Yee-Ling and Shun-Wan, 2014). By inducing nitric oxide production and acting on the insulin signaling pathways, ACH09 (grape skin extract) was found to be extremely effective against obesity, hypertriglyceridemia, hyperglycemia, insulin resistance, and oxidative stress (Shanmuganayagam et al., 2012). According to Angela C et al., grape consumption may lower blood pressure and plasma ROS levels, reduce atherosclerotic plaque formation, and maintain normal serum AST levels (Resende et al., 2013). Several studies have found that grapeseed and grape skin extracts have anti-diabetic effects, improving glucose tolerance and insulin sensitivity in diabetic patients (Chis et al., 2009; Yanni et al., 2015). In the diet-induced obesity mouse models, ACH09 restored decreased plasma and mesenteric artery antioxidant activities of superoxide dismutase, catalase, and glutathione peroxidase (Shanmuganayagam et al., 2012). Several studies have shown that resveratrol slows progression and reduces mortality in CKD rat models, which is attributed to its inhibitory effect on oxidative stress (Byon et al., 2008). Polyphenolic flavonoids derived from grape seeds have been shown to reduce lipid peroxides and carbonylated protein levels in Wistar albino rats. The grape extract increased antioxidant activity in plasma and liver tissues, helping to regulate blood lipids, protect liver cells, and improve blood glucose (Yanni et al., 2015) and suggesting that GENs can improve VC by reducing oxidative stress.

### Inflammation

Chronic inflammation is known to contribute to VC. When monocyte-derived macrophages are recruited and activated in mineralized areas, mineral deposition is triggered (Koji et al., 2012). Excess production of proinflammatory factors such as TNF-α, IL-1, and IL-6 can accelerate the formation of VC by increasing the expression of *BMP2* and decreasing the expression of *MGP* (Koji et al., 2012; Kay et al., 2016). Boström Kristina et al. found that TNF-α, L-1β, and TGF-β can induce and promote endothelial-to-mesenchymal transition, sensitizing aortic endothelial cells to *BMP-9*-induced osteogenic differentiation and enhancing BMP-9-induced mineralization (Kristina, 2005).

GENs were found to inhibit intestinal inflammation in a mouse model of DSS-induced colitis (Songwen et al., 2013). A near-infrared fluorescent dye, DiR, was used to trace the *in vivo* distribution imaging in mice; it was found that GENs can be absorbed by intestinal stem cells by penetrating the intestinal mucosal barrier through the *Wnt/catenin* signaling pathway, stimulating Lgr5hi intestinal stem cell proliferation, accelerating the regeneration of small intestinal mucosa, and promoting rapid recovery of intestinal structure. *Axin-2*, *cyclin D1*, *c-myc*, and *EGFR* expression were found to be significantly upregulated in dextran sulfate sodium (DSS)-induced colitis. It was found that oral administration of GEN protects mice from DSS-induced colitis compared to the PBS control. Orally administered edible plant GENs induce nuclear translocation of macrophage nuclear factor-erythroid-derived 2-related factor-2(*Nrf2*) and intestinal *Wnt/TCF4* activation in mice. Nuclear translocation of *Nrf2* and *Wnt/TCF4* activation are important in anti-inflammatory responses (Songwen et al., 2013).

Furthermore, by inhibiting nuclear factor kappa-light-chain-enhancer of activated B cell (*NF-ҡB*) activation and *COX-2* expression, resveratrol and other biologically active substances can reduce pro-inflammatory cytokines, *PGE2* and *PGD2* levels, and neutrophil infiltration (Cianciulli et al., 2012; Panaro et al., 2012; Gonzalo et al., 2019). Proanthocyanidins were loaded into solid lipid nanoparticles and used to treat inflammatory airway diseases (Baomei et al., 2014). Grape phenolic compounds have anti-inflammatory, anti-cancer, and anti-aging properties (Castellani et al., 2018). A previous study found that grape polyphenols may reduce nitric oxide inactivation *via* oxidant enzymes to prevent inflammation. The main symptoms are lower hypersensitive-c-reactive-protein and IL-6 levels in the blood (Fahimeh et al., 2020). Based on these findings, we hypothesize that GENs lower VC by inhibiting chronic inflammation.

### Apoptosis

Apoptosis of VSMCs occurs prior to the formation of calcified nodules. Proudfoot D et al. reported that inhibiting apoptosis with the caspase inhibitor z-VAD-FMK reduced calcification in nodules by approximately 40%, but when apoptosis was stimulated in nodular cultures with anti-Fas IgM, calcification was increased 10-fold (Proudfoot et al., 2000). Furthermore, they found that apoptotic VSMCs can produce matrix vesicles and apoptotic bodies, both of which have the ability to concentrate calcium and act as nucleating structures for calcium crystal formation (Proudfoot et al., 2000). They found that apoptosis occurs before calcification and that it activates the early promoter in VC.

Grape proanthocyanidins have been found to inhibit H_2_O_2_-induced apoptotic signaling, which is mediated by *p53* in osteoblastic MC3T3-E1 cells (Zhang et al., 2014). Grapeseed proanthocyanidins have been shown to reduce stress-induced apoptosis in the endoplasmic reticulum (ER). Another study found that GSP improved long-term neurological outcomes by reducing ischemia–reperfusion-induced neuronal apoptosis and brain injury and inhibiting the expression of ER stress-associated genes. GSP protects mice against ischemic stroke by reducing neuronal apoptosis and ER stress-associated apoptosis by inhibiting *GRP78* and *caspase-12* (Kun et al., 2019; Yunxia et al., 2020). Thus, it is possible that VC can be improved with GENs by decreasing VSMC apoptosis.

### Other possible mechanisms

An epidemiological study found that osteoporosis and VC have age-independent associations (Yunxia et al., 2020; Hak et al., 2000). Thandapilly Sijo J et al. discovered CKD patients with dysregulated calcium and phosphate metabolism. However, there are some issues to consider in the relationship between calcium loss from the skeleton in osteoporosis and calcium deposits in VC (Tofani et al., 2005; Demer et al., 2014; Wei S. et al., 2018). Grapeseed proanthocyanidin extract induces anti-osteoporosis effects by increasing bone mineral density and bone strength (Tofani et al., 2005; Wei Z. et al., 2018).

Furthermore, GENs containing some miRNAs can be uptake by VSMCs to participate in immunoregulation. Grape flavonoids can regulate endothelial function and improve endothelial-dependent vasodilation in the aorta (Stein et al., 1999). Numerous molecular targets (silent information regulator 1 (*SIRT1*), 5′ AMP-activated protein kinase (*AMPK*), endothelial nitric oxide synthase (*eNOS*), *Nrf2*, peroxisome proliferator-activated receptor (*PPAR*), Kruppel-like factor 2 (*KLF2*), and *NF-kB*) (Nader et al., 2021) were found. Several recent studies have found that polyphenol extracts from grapes provide cardiovascular benefits by lowering blood pressure, improving the relaxation of arterial smooth muscle, increasing arterial compliance, and attenuating pathological cardiac hypertrophy (Thandapilly et al., 2012). This approach is expected to be used in the treatment of VC.

## Conclusion

VC is a key factor in the development of cardiovascular disease. Although the development of therapeutics for the treatment of VC in experimental treatment has made great progress, clinical first-line medication is still lacking. Unfortunately, current anti-VC drugs are primarily based on phosphate binders and calcimimetic agents, which are restricted by inefficient drug delivery and short residence time (Lanzer et al., 2021; Raggi et al., 2011). GENs are expected to serve as novel drug carriers with synergistic effects for the delivery of anti-VC drugs due to their high biocompatibility, low immunogenicity, and easy modifiability (Songwen et al., 2013).

**FIGURE 2:**
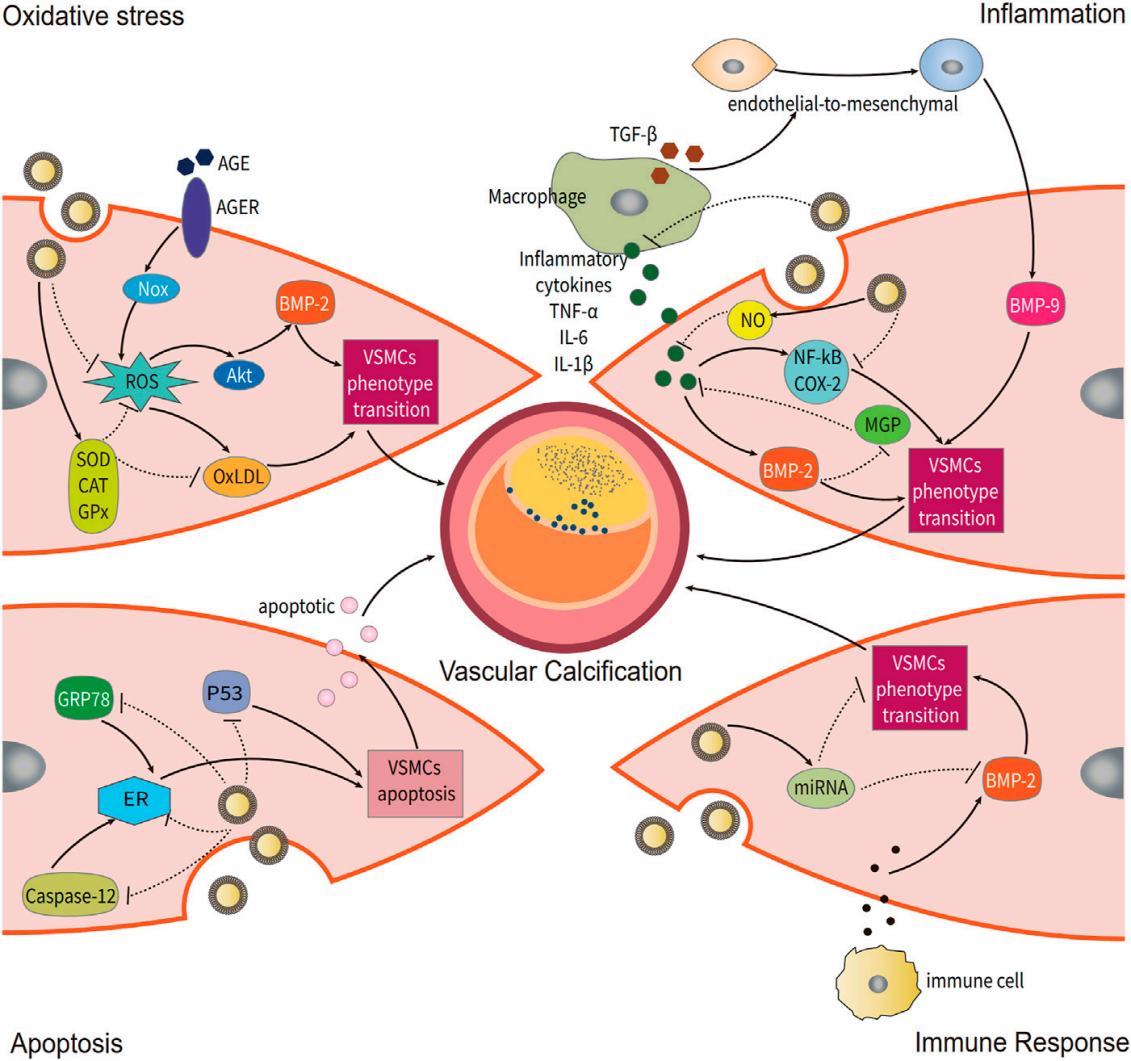
Potential regulatory mechanisms of grape exosome-like nanoparticles (GENs) for vascular calcification (VC). Several mechanisms of VC occurrence are modulated by GENs, including oxidative stress, inflammation, apoptosis, and immune response.

In this review, we discussed the potential treatment efficacy and various mechanisms of GENs related to affecting the VC (Figure 2). Despite the many benefits of GENs, we still need to consider the following deficiencies in the treatment of VC: 1. particle size distribution is relatively nonuniform; larger particles might not penetrate the intercellular space of vascular endothelium and enter calcified plaques. 2. The complete removal of plant-derived impurities cannot be guaranteed in the process of isolation and purification, causing immune responses. 3. The roles and functions of GENs against VC are unclear, which still is a hypothesis based on existing knowledge of VC and GENs. Therefore, they could have unpredictable effects (Kim et al., 2022). Overall, GENs demonstrate a potential protective effect on VC, and they may constitute the next-generation therapeutics hopefully.

## Abbreviations

CKD, chronic kidney disease; DiR, 1,1-dioctadecyl-3,3,3,3-tetramethylindotricarbocyaine iodide; DSS, dextran sulfate sodium; ER, endoplasmic reticulum; GEN, grape exosome-like nanoparticle; GNV, grapefruit-derived nanovector; HA, hydroxyapatite; OxLDL, oxidized low-density lipoprotein; PDEN, plant-derived exosome-like nanoparticle; ROS, reactive oxygen species; VC, vascular calcification; VSMC, vascular smooth muscle cell.
